# Comparative Analysis of Color Stability and Its Impact on Artificial Aging: An In Vitro Study of Bioactive Chitosan, Titanium, Zirconia, and Hydroxyapatite Nanoparticle-Reinforced Glass Ionomer Cement Compared With Conventional Glass Ionomer Cement

**DOI:** 10.7759/cureus.54517

**Published:** 2024-02-20

**Authors:** Dhivya Sri E, Jessy Paulraj, Subhabrata Maiti, Rajeshkumar Shanmugam

**Affiliations:** 1 Department of Pedodontics and Preventive Dentistry, Saveetha Dental College and Hospitals, Saveetha Institute of Medical and Technical Sciences, Saveetha University, Chennai, IND; 2 Department of Prosthodontics, Saveetha Dental College and Hospitals, Saveetha Institute of Medical and Technical Sciences, Saveetha University, Chennai, IND; 3 Nanobiomedicine Lab, Centre for Global Health Research, Saveetha Medical College and Hospital, Saveetha Institute of Medical and Technical Sciences, Chennai, IND

**Keywords:** bioactive nanoparticles, gloss, thermocycling, nano modified gic, color stability

## Abstract

Background

Discoloration affects glass ionomer cement (GIC) color stability due to its brittle nature and microporosity. To counter this, incorporating alternative materials is essential for maintaining color stability.

Aim

This study aims to determine the color stability and gloss of GIC modified with bioactive chitosan, titanium, zirconia, and hydroxyapatite nanoparticles before and after artificial aging.

Materials and methods

The study was conducted at Saveetha Research Centre, Saveetha Dental College and Hospitals, Saveetha Institute of Medical and Technical Sciences, Saveetha University, located in Chennai, India. Green-mediated chitosan, titanium, zirconia, and hydroxyapatite (Ch-Ti-Zr-HA) nanoparticles were synthesized using the one-pot synthesis technique. Forty-eight disc-shaped specimens were prepared by incorporating the obtained nanoparticles (nanocomposite) into the GIC, with a diameter of 5 mm and thickness of 2 mm. The specimens were prepared in different concentrations (3%, 5%, and 10%) designated as group I, group II, and group III, respectively. Group IV, serving as the control, consisted of conventional GIC without any modifications. Following preparation, scanning electron microscopy (SEM) and energy-dispersive X-ray (EDX) microanalysis confirmed sample elements, and the specimens were submerged in distilled water for a duration of 24 hours prior to the commencement of testing. Subsequently, the specimens underwent artificial aging (thermocycling), between temperatures of 5°C and 55°C, for a total of 30,000 cycles, with a 30-second dwell time. Color change and gloss characteristics were assessed both after 24 hours and following thermocycling using a spectrophotometer and glossometer, respectively. The average color change parameter (ΔE) was measured using Adobe Photoshop. The data obtained were subjected to statistical analysis using an unpaired t-test.

Results

Significant color stability variations were observed post thermocycling (P = 0.001). Group 2 (5%) exhibited minimal delta E difference (0.508 ± 0.105), indicating superior color stability, while group 4 (control) had maximum difference (1.15 ± 0.187), indicating lower stability. Gloss tests confirmed GIC's polishability, where there were significant differences among all the groups.

Conclusion

It can be concluded that 5% nanoparticle-modified GIC has better color stability and gloss than conventional GIC. Further studies are needed to analyze the color stability and gloss through dentifrices and other beverages.

## Introduction

The need for attractive dental restorations has considerably increased in recent times, prompting the development of a variety of materials [1]. Glass ionomer cement (GIC), as the forerunner among aesthetic restorative materials, has been a significant player owing to its capacity to release fluoride and form a chemical bond with the tooth. GIC has traditionally been the favored option, initially applied in anterior teeth and suggested for class III and V restorations. Its advantageous feature lies in its capacity for physicochemical binding to enamel and dentin through ion exchange mechanisms, releasing fluoride and other ions that establish a strong connection between the tooth structure and GIC [2]. However, GIC is characterized by brittleness, leading to unfavorable mechanical properties, and this brittleness contributes to the presence of high microporosity in GIC. The microporosity in GIC can influence its capacity to preserve color stability [2]. Moreover, challenges arise for dental professionals and manufacturers in the oral environment, where GIC, similar to other restorative materials, encounters exposure to saliva, oral microflora, and frequent intake of colored food and beverages.

The physicochemical properties of GIC, particularly the conventional variant, play a critical role during the early setting stages and are notably affected by the surrounding conditions. Several studies suggest that conventional GIC exhibits substantial color changes, particularly in acidic conditions, compared to other aesthetic restorative materials [3,4]. Efforts have been made to address these limitations and enhance the mechanical properties and color stability of GIC through modifications. Earlier studies attempted to augment the glass powder with additions, like metallic oxides, strontium, and barium; however, these proved ineffective in significantly improving the mechanical properties and color stability of GICs due to their inability to enhance cross-linking within the glass matrix [5]. The emergence of nanotechnology in the early 21st century opened new avenues for reinforcing GICs [6]. Recent investigations have demonstrated that the inclusion of nanoceramics, including hydroxyapatite (HA) and zirconia (ZrO_2_), synthesized through diverse soft chemistry methods to yield nanoscale particles, show promise in improving the characteristics of GICs [7].

Color stability stands as a pivotal element directly influencing the clinical efficacy of aesthetic restorative materials. The comprehensive aesthetics of restorations include facets, like color, translucency, and opacity, along with light reflectance, transmittance, and surface texture [1]. The measurement of dental restorative material color involves tools, like shade guides, colorimeters, and spectrophotometers. Among the various techniques for identifying color alterations, the application of the L ∗ a ∗ b axis within the CIELAB (Commission Internationale Declairage) system has been established as the most favored [8]. This system allows for reliable, quantifiable, and reproducible documentation of color changes. Achieving an optimal gloss is a critical aspect of dental aesthetics, as it contributes significantly to the overall appearance and perceived quality of dental restorations. The gloss of these materials is not only an indicator of their surface smoothness but also influences how light interacts with the restoration, impacting its visual integration with surrounding natural teeth [8]. Dental professionals strive to select and manipulate restorative materials to attain the desired gloss, ensuring that the resulting restorations seamlessly blend with the patient's dentition while providing long-lasting functional and aesthetic benefits.

GICs manifest varying clinical deterioration, even in the absence of mechanical stress. This degradation is ascribed to thermal shocks in the oral environment, causing the disintegration of metal-polyacrylate bonds in GICs [9]. This degradation may lead to potential color alterations in these materials. Thermocycling offers a way to simulate the behavior and performance of dental restorative materials during clinical service to some extent. In thermocycling, materials are subjected to repeated temperature changes that mimic those occurring frequently in the oral cavity [10]. Evaluating dental restorative materials through thermocycling provides a more accurate simulation of their performance during clinical use. Moreover, as age-related studies are insufficient, there is a need to understand the shelf life of the material, so the objective of the study was to assess the color stability and gloss of green-mediated nanoparticles, chitosan, titanium, zirconia, and hydroxyapatite (Ch-Ti-Zr-HAP) incorporated GIC over conventional GIC before and after thermocycling. The null hypothesis posited that the incorporation of nanoparticles into GIC would not influence color stability compared to conventional GIC following the aging process.

## Materials and methods

Estimation of sample size and study design

The study was conducted at Saveetha Research Centre, Saveetha Dental College and Hospitals, Saveetha Institute of Medical and Technical Sciences, Saveetha University, located in Chennai, India. Ethical approval was obtained for this in-vitro study from the Institutional Review Board (SRB/SDC/UG-1994/23/PEDO/135). Sample power calculations were conducted utilizing the GPower sample power calculator. The calculations revealed that, in order to achieve a sample power of 0.95 (with a 95% confidence interval) and an effect size of 0.8, each group was required to have 48 samples.

Preparations of green-mediated nanoparticles (Ch, Ti, Zr, HAP)

To produce plant-derived nanoparticles, 50 ml of 1 gm eucalyptus was blended with 50 ml of chitosan (0.5 g chitosan powder mixed with 0.5 g glacial acetic acid and 49 ml distilled water) using a magnetic stirrer, resulting in plant-based chitosan nanoparticles. Similarly, 50 ml of 1 gm neem was stirred with 50 ml of 50 millimolar TiO_2_ using a magnetic stirrer to generate plant-based titanium oxide nanoparticles. For plant-based zirconium oxide nanoparticles, 50 ml of aloe vera was added to 50 ml of 20 millimolar zirconium oxide, continuously agitated at 340-350 °C with a magnetic stirrer, and stored overnight. Lastly, 50 ml of 1 gm *Moringa oleifera* was mixed with 50 ml of 0.1 gm hydroxyapatite (synthesized from eggshell), stirred continuously, and orthophosphoric acid was added drop by drop, maintaining a molar ratio of 1.67 Ca/P, followed by overnight stirring, resulting in plant-based hydroxyapatite nanoparticles.

Preparation of green-mediated nanocomposite (Ch-Ti-Zr-HAP)

Utilizing the one-pot synthesis method described by Rehman et al. [11], the four final solutions (chitosan, titanium, zirconium, and hydroxyapatite) were combined, vigorously stirred at 80 °C for 30 min, and ethanol (1.08 mL) was added to the main batch. After vigorously stirring under reflux conditions at 80 °C for 90 minutes, the reaction vessel was subsequently opened and maintained at 80 °C for an additional 30 minutes to remove ethanol. The resulting solution was lyophilized in a freeze dryer for 48 hours at -92˚C and finely powdered to obtain the final product of nanocomposites. Lyophilization was conducted to enhance the long-term durability of nanoparticles, ensuring that the biochemical properties of the active ingredient remain unchanged due to the gentle freeze-drying process.

Preparation of green-mediated nanocomposite (Ch-Ti-Zr-HAP) modified GIC

The green-mediated (Ch-Ti-Zr-HA) nanocomposites were incorporated into GIC in 3%, 5%, and 10% into the powder component of GIC as group I, group II, and group III, respectively, and group IV, with conventional restorative GIC (control), and then the powder component was mixed using the polyacrylic acid based liquid to form a restorative cement.

Specimen preparation 

For each group, 48 disc-shaped specimens were prepared. The powder and liquid components of the materials were measured by weight using a Mettler AJ100 balance (Greifensee, Switzerland) on a paper mixing pad. Green-mediated nanocomposite (Ch-Ti-Zr-HA) was incorporated into GIC at concentrations of 3%, 5%, and 10%. The blending was carried out using the polyacrylic acid-based liquid supplied by the manufacturer (GC Corporation), following the provided instructions. The components were mixed with a plastic spatula until a uniform paste was achieved. This mixture was then placed into a split metal mold with a diameter of 5 mm and a thickness of 2 mm. Following the setting process, the samples were retrieved and the specimens were examined under a bright light source to identify any porosities or cracks. Specimens with variations in porosity or cracks were excluded from the study. Every sample underwent finishing and polishing before being stored at room temperature.

Scanning electron microscopy (SEM) and energy-dispersive X-ray (EDX) microanalysis

**Figure 1:**
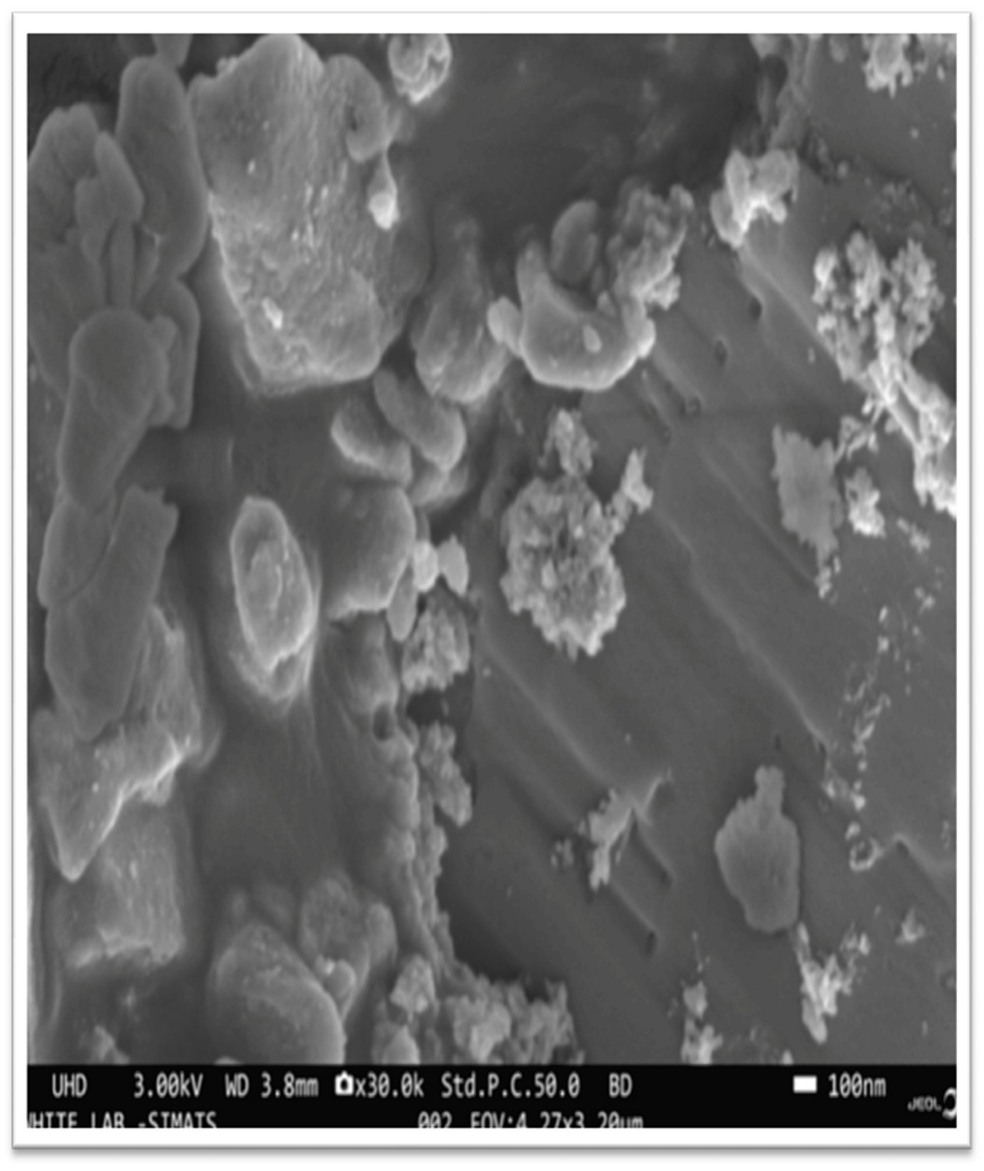
Scanning electron microscopy (SEM) analysis

Nanoparticle confirmation was conducted using a combination of scanning electron microscopy (SEM) and energy-dispersive X-ray (EDX) microanalysis (Figure 1).

**Figure 2:**
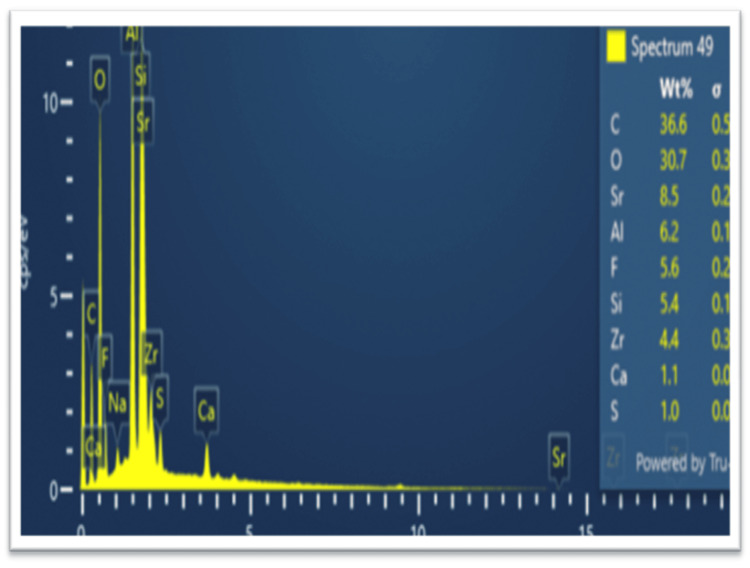
Energy-dispersive X-ray (EDX) microanalysis

EDX microanalysis involves detecting and examining X-rays emitted by a specimen when exposed to an electron beam. This method provides essential details about the elemental composition of the sample, enabling the identification and quantification of elements within the analyzed region. Often used in conjunction with SEM, EDX microanalysis offers insights into the elemental constitution of materials at the microscale. The resulting spectra from EDX analysis display peaks corresponding to the energy levels of emitted X-rays, aiding in identifying specific elements present in the sample. This analytical approach proved invaluable for exploring the elemental makeup of cutting-edge nanocomposites (Figure 2).

Thermocycling of the specimens

**Figure 3:**
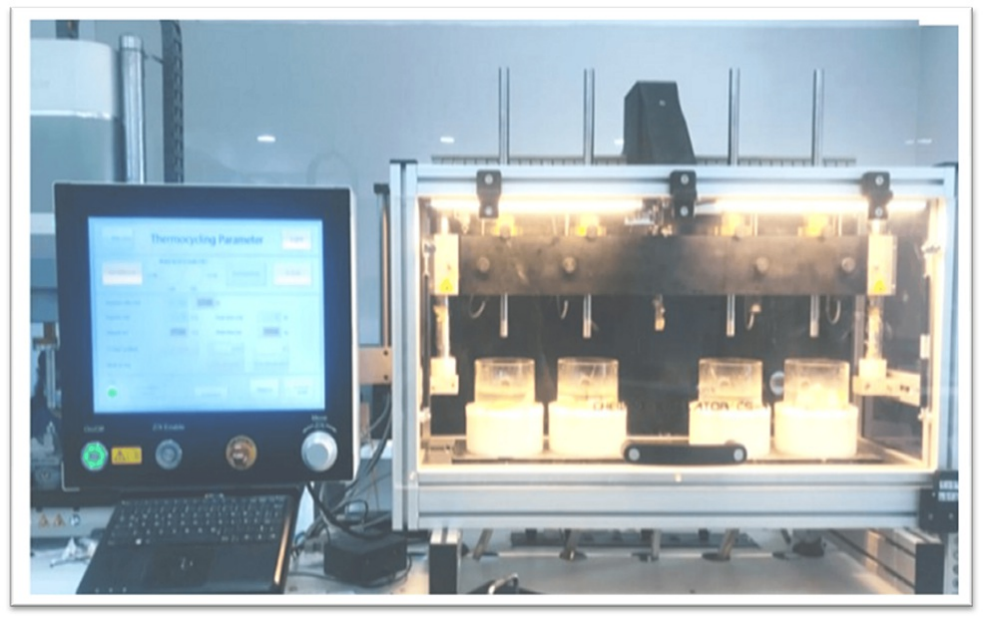
Thermocycling

The chosen specimens were immersed in distilled water for a duration of 24 hours before undergoing testing to prevent discoloration due to external factors, prior to the first color evaluation. Thermocycling was performed employing a thermocycler (Lab Thermostatic Bath) that housed water baths held at a constant temperature of 55 °C. The specimens experienced thermocycling between 5 °C and 55 °C, undergoing a total of 30,000 cycles, each with a dwell time of 30 seconds (Figure 3).

Color measurement

**Figure 4:**
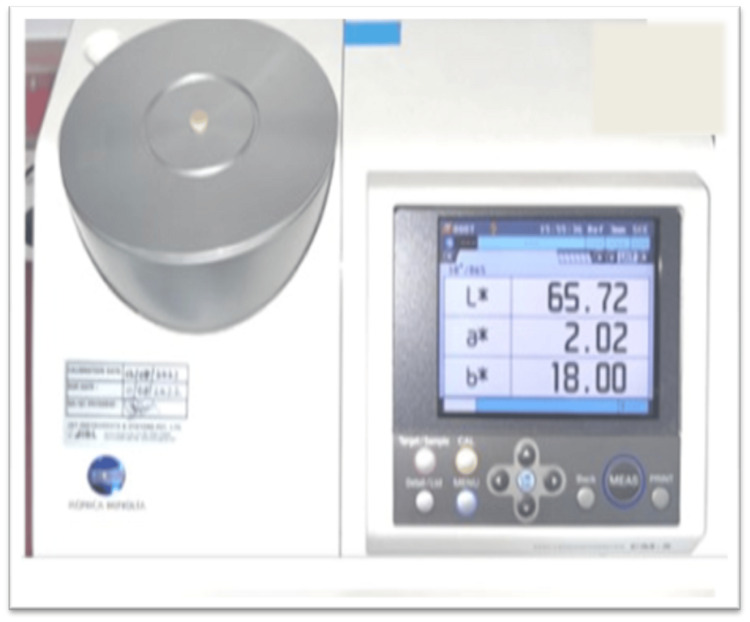
Color analysis using a spectrophotometer

The optical properties were assessed using a computer-controlled spectrophotometer, which provided color coordinates in the CIELAB system. This system includes L* (representing lightness with 100 as white and 0 as black), a* (indicating the red-green chromaticity index), and b* (indicating the yellow-blue chromaticity index) against a standard white background. Throughout the testing period, samples were stored in airtight containers at 37 °C and 100% relative humidity. The CIE L*, a*, b* values of the experimental groups (group I, group II, and group III) with a green-mediated nanocomposite (Ch-Ti-Zr-HA) were compared with the conventional GIC group (group IV) to assess color change after 24 hours before thermocycling and after thermocycling (30,000 cycles). The color stability was evaluated by calculating the delta-E difference using a delta-E calculator before and after thermocycling (Figure 4).

The color alterations in the specimens were assessed by utilizing the subsequent formula:

ΔEab(L*a*b) = [ (ΔL*)2 +(Δa*)2 +(Δb*)2]1/2

The ΔE value indicates the extent of color change: 0-2 signifies no observable color change, 2-3 indicates a slight change, and 3-8 or higher suggests a noticeable color change. Values of ΔE equal to or below 3.7 are considered clinically acceptable [12].

Determination of gloss 

**Figure 5:**
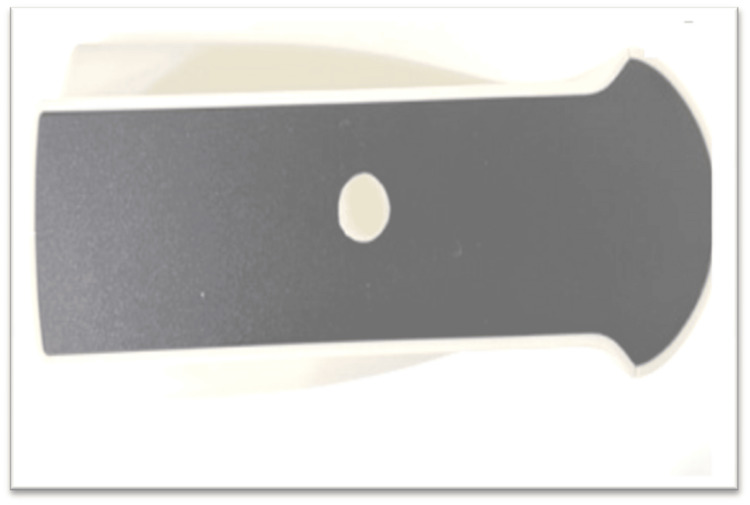
Gloss analysis by a gloss meter

Quantitative information on a material's reflective properties is obtained through gloss meter measurements, revealing how light interacts with the surface. The process entails projecting a beam of light onto the surface at a fixed intensity and angle and then measuring the reflected light at an equivalent but opposite angle. Gloss was assessed using a gloss meter (3 nh) before and after thermocycling for each sample (Figure 5).

Statistical analysis

The gathered data were inputted into a Microsoft Excel spreadsheet and underwent statistical analysis using IBM SPSS Statistics for Windows, version 24 (released 2016; IBM Corp., Armonk, New York, United States). The color changes among the groups were assessed through one-way ANOVA, with subsequent pairwise comparisons conducted using the Tukey test. In addition, a paired t-test was employed to evaluate differences between pre- and post-thermocycling conditions.

## Results

Color stability was assessed based on the difference of delta E values between pre and post thermocycling. A significant difference (P = 0.001) in color stability was found among all groups (P < 0.05), where group 2 (5%) showed a minimum (0.508 ± 0.105) difference indicating maximum color stability and group 4 (control) showed (1.15.± 0.187) a maximum difference of delta E value indicating minimum color stability (Table 1).

**Table 1:** Comparison among groups based on changes in color (delta E value from the CIELAB color measuring system) between pre and post thermocycling *statistically significant value of p < 0.05; SD = standard deviation, SE = standard error, CI = confidence interval P-value was derived from a one-way analysis of variance (ANOVA) test.

	Groups	Mean ± SD	SE	95% CI		F value	P value	Null hypothesis
				Lower	Upper			
Color stability	Group 1 (3%)	0.840±0.072	0.018	0.800	0.880	67.195	0.001*	Rejected
	Group 2 (5%)	0.508±0.105	0.027	0.450	0.566			
	Group 3 (10%)	0.926±0.109	0.028	0.866	0.987			
	Group 4 (control)	1.15±0.187	0.048	1.047	1.254			

The gloss test indicated the polishability and glossy appearance of GIC. A notable distinction was observed in both pre-thermocycling (P = 0.001) and post-thermocycling (P = 0.001) values among all groups and mean difference between pre and post thermocycling (P = 0.001) (Table 2).

**Table 2:** Comparison among groups based on change in gloss between pre and post thermocycling *statistically significant value of p < 0.05; SD = standard deviation, SE = standard error, CI = confidence Interval. P-value was derived from a one-way analysis of variance (ANOVA) test.

	Gloss test: Pre thermocycling		Gloss test: Post thermocycling		Gloss difference	
Group	Mean ± SD	P value	Mean ± SD	P value	Mean ± SD	P value
							Group 1 (3%)	38.43 ± 1.25	0.001*	51.08 ± 2.23	0.001*	12.6 ± 3.09	0.001*
Group 2 (5%)	41.70 ± 0.81	58.57 ± 1.65	16.86 ± 1.67										
Group 3 (10%)	29.58 ± 2.30	37.08 ± 5.54	7.49 ± 5.72										
Group 4 (control)	41.34 ± 1.49	49.12 ± 0.80	8.44 ± 2.95										

On pairwise comparison, no difference was found between the groups except between 3% and 10%, where 10% showed the maximum variation after thermocycling (Table 3).

**Table 3:** Pairwise comparison between groups based on the change in color (delta E value from the CIELAB color measuring system) and gloss between pre and post thermocycling *statistically significant value of p < 0.05; MD = mean difference, CI = confidence interval. P-value was derived from Tukey’s post-hoc analysis.

	Groups	MD	P value	95% CI	
						Lower	Upper
				Color stability (difference of delta E value)	Control vs. 3%	0.310	0.001*	0.188	0.431
Control vs. 5%	0.642	0.001*	0.520		0.763
Control vs. 10%	0.224	0.001*	0.102		0.345
3% vs. 5%	0.332	0.001*	0.210		0.453
3% vs. 10%	0.086	0.252	0.035		0.207
5% vs. 10%	0.418	0.001*	0.296		0.539
Gloss test (difference of gloss value )	Control vs. 3%	4.15	0.016*	0.601	7.70
	Control vs. 5%	8.42	0.001*	4.87	11.97
	Control vs. 10%	0.94	0.894	-2.60	4.49
	3% vs. 5%	4.26	0.012*	0.717	7.82
	3% vs. 10%	5.10	0.002*	1.54	8.65
	5% vs. 10%	9.37	0.001*	5.81	12.92

## Discussion

In the contemporary landscape of highly aesthetic dentistry, patients seek restorations that not only meet aesthetic standards initially but also maintain their color and appearance over an extended period. This demand has prompted the development of various materials to address these requirements [11]. GIC, as a forerunner in aesthetic restorative materials, is commonly utilized in aesthetic procedures, especially for primary teeth, owing to its capacity for fluoride release and chemical bonding with the tooth [12]. However, its shortcomings, such as low strength, limited abrasion resistance, moisture sensitivity, and porosity, have compromised aesthetics, prompting the search for newer and improved materials. The incorporation of additives has been noted to influence the color stability of glass ionomers [13]. However, the extent and clinical significance of this color instability vary depending on the type of nanoparticle used. In addition, many traditional physical and chemical techniques for nanoparticle synthesis involve the use of harmful chemicals. Therefore, an environmentally friendly green synthesis technique was employed to produce nanoparticles in this study. All dental cement exhibited different levels of discoloration after accelerated aging, prompting an evaluation of the newer restorative material both before and after thermocycling.

The ever-changing conditions within the oral environment, characterized by variations in pH, stress, and temperature, can significantly influence the color stability of aesthetic restorative materials. The current investigation revealed substantial alterations in the color characteristics of the materials after thermocycling, particularly at the 30,000-cycle mark. In this research, the CIELAB color system was employed, aligning with the recommendations of the American Dental Association. In this study, the color stability of nanocomposite-modified GIC has shown better results compared to conventional GIC. In particular, 5% nanocomposite-modified GIC (group II) exhibited a significantly lower mean delta E value compared to the other groups (p < 0.05), consistent with the findings of Sami et al. [14]. Their study suggested that nanoZrO_2_-SiO_2_-HA significantly enhances color stability, positioning it as a recommended restorative dental material, aligning with our study [14]. Other studies have also highlighted lower color stability in conventional GIC, supporting our present investigation. Ahmed et al. indicated that resin-modified GIC shows improved color stability compared to conventional GIC [15,16]. A study done by Bhattacharya and et al. concluded that cention performed better color stability when compared to conventional GIC [1]. By contrast, Pani et al. reported that the inclusion of strengthening materials leads to a notable reduction in the color stability of glass ionomer cement [17]. Another study by Bajpai et al. presented a contrasting view, suggesting that the handling and mixing involved in the preparation of a material, particularly with powder and liquid components, pose an elevated risk of incorporating air bubbles. This increased porosity may result in higher surface roughness and greater pigment retention, leading to discoloration [18-20]. In this study, zirconia and chitosan present in the nanocomposite could have produced a mechanical interlocking phenomenon resulting in zero or minimal porosity and hardening. This effect would make the ionomer less hydrolytic and more stable to color change and confer good chemical resistance in the oral environment. In addition, one of the proposed benefits of utilizing nanoparticles is that, due to their small size, it becomes feasible to uphold recommended powder-to-liquid ratios while still obtaining a durable matrix [21-23].

Glossometer measurements offer quantitative insights into a material's reflective properties, revealing how light interacts with its surface. In dental material research, particularly concerning restorative materials like GIC, such studies are crucial, as surface gloss plays a significant role in the aesthetics of dental restorations. The investigation delved into how factors, such as moisture, wear, and exposure to oral conditions, affect the gloss of GIC, considering its clinical application as a dental restorative material. The findings indicated that GIC undergoes changes over time, especially after exposure to various environmental factors like thermocycling, resulting in a loss of the glossy surface in conventional GIC compared to nanocomposite-modified GIC groups. This difference can be attributed to nanoparticle incorporation, which contributes to a more polished and glossy surface.

All the modified GIC showed better color stability than conventional GIC, where the 5% nanocomposite-modified GIC showed maximum color stability, followed by the 3% and 10% nanocomposite-modified GIC. In terms of gloss, the 10% nanocomposite-modified GIC showed maximum gloss, followed by the 5% control and 3% nanocomposite-modified GIC. The glossy surface of the GIC mix signifies the existence of residual polyacid, which is not utilized in the setting reaction but aids in adhesive bonding to the tooth. Furthermore, GIC lacks color stability and is not as robust as metallic restorations. Evidence indicates that GIC is brittle and prone to cracking due to its low flexural strength. To provide more enduring treatments for patients, green-mediated nanocomposite (Ch-Ti-Zr-HAP)-modified GIC emerges as a preferable option due to its improved properties, making it suitable for both anterior and posterior restorations with superior color stability than conventional GIC restorations. Thus, the null hypothesis is rejected, proving that color stability and gloss are enhanced for green-mediated nanocomposite GIC when compared with conventional GIC.

It is important to recognize as a limitation that the present in vitro study does not allow for a completely accurate prediction of the obtained results in a clinical setting. This is due to various factors influencing the color stability of restorative materials within the oral cavity, such as the salivary film and the effects of specific foods, which are challenging to replicate in an in vitro environment. Further research is advisable to investigate material discoloration, considering various polishing techniques, surface roughness, and protective coatings at different immersion durations, which can aim to develop advanced restorative materials that address the current limitations of the present study.

## Conclusions

Within the limitations of this study, it can be concluded that the 5% modified GIC demonstrated superior color stability and gloss compared to conventional GIC formulations. These findings suggest the potential for broader utilization of the green-mediated nanocomposite (Ch-Ti-Zr-HA), particularly in anterior aesthetic restorations. The enhanced stability and gloss offered by this modified GIC could significantly improve the longevity and aesthetic outcome of dental restorations, contributing to patient satisfaction and clinical success. Future research endeavors are necessary to rectify the limitations and advance our understanding of the properties and effectiveness of GIC.
